# Beyond exhaustion: T cell fitness for next generation of immunotherapy for hematological cancer

**DOI:** 10.3389/fimmu.2026.1895434

**Published:** 2026-07-27

**Authors:** Xiaoshuang Kong, Yuanhui Su, Hanxue Zheng, Zuxi Feng, Jianxia Huang, Liansheng Zhang, Lijuan Li

**Affiliations:** 1 Department of Hematology, The Second Hospital & Clinical Medical School, Lanzhou University, Lanzhou, China; 2 Gansu Province Clinical Medical Research Center for Blood Diseases, Lanzhou, China; 3 The Second Hospital & Clinical Medical School, Lanzhou University, Lanzhou, China; 4 Wuwei People’s Hospital Wuwei, Wuwei, China; 5 Department of Hematology, The Second Hospital of Lanzhou University, Lanzhou, China

**Keywords:** biomarkers, bispecific antibodies, CAR-T cell therapy, hematological malignancies, t cell exhaustion, T cell fitness, T cell senescence, treatment sequencing

## Abstract

T cell-directed immunotherapies have transformed the treatment of hematological malignancies, but durable benefit remains limited by relapse, poor persistence, incomplete immune reconstitution and infection. These outcomes depend not only on target-antigen expression but also on the functional quality of the T cell compartment. In this Review, we define T cell fitness as a multidimensional capacity that includes cellular availability, memory reserve, proliferative competence, cytotoxic function, metabolic resilience, resistance to chronic stimulation and persistence. We distinguish exhaustion, senescence and terminal differentiation as overlapping but non-equivalent states and propose a measurable, modality-specific assessment framework rather than reliance on a single marker. We then compare how these states arise in multiple myeloma, lymphoma, acute lymphoblastic leukemia and acute myeloid leukemia, emphasizing the effects of age, tissue niche, disease burden and prior therapy. We critically appraise evidence from CAR-T cell therapy, bispecific antibodies and checkpoint blockade, including the limitations of predominantly retrospective, correlative and disease-specific datasets. Finally, we translate the fitness framework into clinical questions: when to collect cells, how to select bridging therapy, which biomarkers merit prospective testing, how treatment duration and sequencing may preserve immune competence, and where gene-edited products, CAR-NK cells and metabolic interventions may reduce dependence on compromised autologous T cells. A fitness-based approach may support more precise biomarker development and safer treatment selection, but prospective validation and modality-specific thresholds are still required.

## 1 Introduction

T cell-directed immunotherapies have redefined outcomes in hematological malignancies. CD19-directed CAR-T cells (1, 2), BCMA-directed CAR-T cells, bispecific antibodies and immune checkpoint inhibitors can induce deep responses in otherwise refractory disease (3–8). Importantly, bispecific antibodies have extended this principle of immune redirection without ex vivo manufacture: blinatumomab improved median overall survival from 4.0 to 7.7 months over chemotherapy in relapsed or refractory B-cell precursor ALL, and teclistamab induced responses in 63% of triple-class–exposed patients with myeloma. Together, these studies established T cell redirection as a major therapeutic platform across B-ALL, lymphoma and myeloma (9–12). Nevertheless, relapse, antigen escape, limited cellular persistence, delayed immune reconstitution and infection remain common barriers. The relevant biological question is therefore no longer only whether a target can be engaged, but whether the recruited or engineered T cells can expand, repeatedly kill, persist and recover safely after treatment (8, 13, 14).

Clinical and translational studies support this view (15, 16). In chronic lymphocytic leukemia, complete response to CD19 CAR-T cell therapy was associated with memory-related programs, whereas nonresponse was associated with effector differentiation, glycolysis, exhaustion and apoptosis (17). In multiple myeloma (MM), T cells collected after initial therapy showed a more favorable memory phenotype and greater ex vivo proliferative capacity than cells collected after multiple relapses (18). Bispecific antibodies expose the same dependency because every dose recruits the endogenous T cell pool; continuous stimulation can progressively impair function, whereas treatment-free intervals can permit partial recovery in experimental systems (19–22). These findings establish T cell quality as a therapeutic substrate, but they do not yet define a universally validated clinical test (23).

The evidence base also has important limitations. Most candidate fitness biomarkers come from small translational cohorts, retrospective analyses, selected trial populations or preclinical models. Marker definitions and sampling time points differ substantially, and associations observed in one disease or therapeutic platform may not generalize to another (24, 25). The contrasting activity of PD-1 blockade in classical Hodgkin lymphoma and its unfavorable benefit-risk profile in MM combinations illustrates why checkpoint expression alone cannot be assumed to identify a reversible state (26–28). A useful framework must therefore be quantitative, disease-aware, therapy-specific and explicit about uncertainty.

MM is an instructive, but not universal, model (29, 30). It combines an older patient population, a suppressive bone marrow niche, chronic antigen exposure and substantial cumulative treatment pressure (31, 32). Botta et al. demonstrated that, during myeloma evolution, large clonal T−cell populations expressing multiple immune checkpoints accumulate, T−cell receptor diversity contracts, and loss of CD27 correlates with clonal expansion and treatment resistance (33). Lymphoma is shaped more strongly by lymph-node architecture, Tregs, macrophages and spatial immune contexture; ALL is dominated by product persistence and antigen escape; and AML combines marrow suppression with target overlap on normal hematopoiesis (34). These differences argue against extrapolating a single exhaustion signature across hematological malignancies (35).

The knowledge gap addressed by this Review is the absence of an integrated framework that links measurable T cell attributes to disease context and to the distinct biological requirements of CAR-T cells, bispecific antibodies and checkpoint blockade (36, 37). We therefore: (i) distinguish exhaustion, senescence and terminal differentiation; (ii) propose a practical multidomain fitness assessment for prospective studies; (iii) compare disease-specific landscapes; (iv) critically evaluate therapy-specific evidence and real-world limitations; and (v) outline clinically actionable questions for leukapheresis timing, bridging therapy, sequencing, treatment duration and next-generation immune engineering.

## 2 Defining T cell dysfunction in hematological cancer: exhaustion, senescence and terminal differentiation

The term “T cell dysfunction” is often applied to any checkpoint-positive or poorly responsive T cell, but this usage obscures states with different origins and reversibility (35, 38–42). CAR-T cell therapy, bispecific antibodies and checkpoint blockade require different combinations of proliferation, cytotoxicity, metabolic resilience and persistence. A clinically useful taxonomy must therefore separate functional impairment from differentiation state and must integrate phenotype with functional and longitudinal evidence.

**Figure 1:**
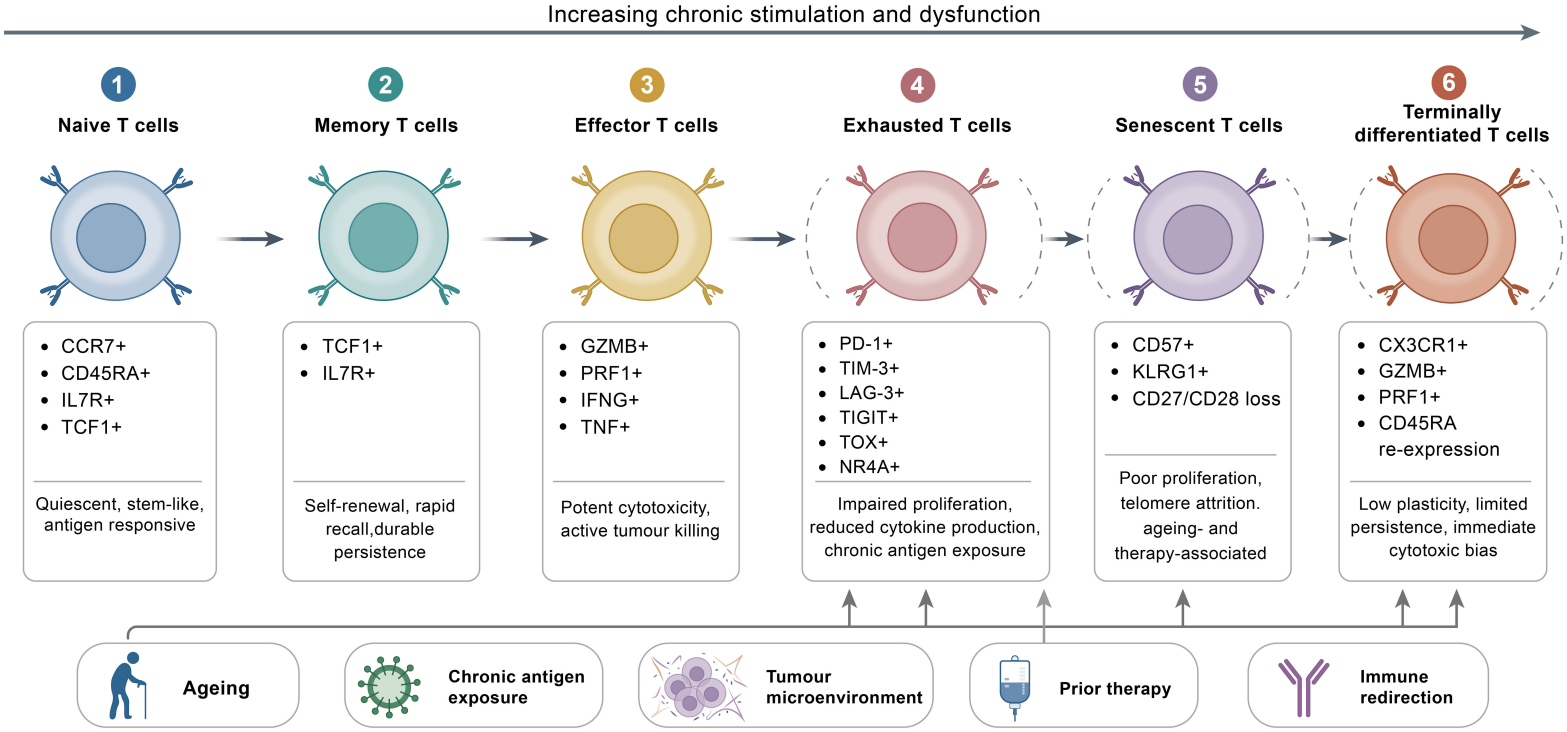
Distinguishing major states within the T cell fitness landscape. Naïve and memory populations provide self-renewal and persistence; effector cells provide immediate cytotoxicity; exhaustion, senescence and terminal differentiation compromise different functions and may overlap. Clinical interpretation: phenotype should be integrated with proliferation, function, clonality and persistence rather than used as a single-state label.

Exhaustion is primarily driven by persistent antigen and is maintained by transcriptional and epigenetic remodeling; senescence reflects replicative history, aging, telomere and DNA-damage stress, and loss of proliferative competence; terminal differentiation describes an effector end state that may retain immediate cytotoxicity but has reduced plasticity and persistence (43–47). These programs can coexist in the same patient or clonotype. Their overlap explains why a single surface marker cannot determine whether dysfunction is reversible or which immunotherapy will be limited (**Figure 1**).

### 2.1 T cell exhaustion

T cell exhaustion was defined in chronic infection as a progressive loss of cytokine production, proliferation and cytolytic function under persistent antigen exposure (48–51). A defining feature of exhaustion is the co-expression of multiple inhibitory receptors. PD-1 alone is insufficient to define exhaustion, as it can also mark recent T cell activation. More definitive exhaustion phenotypes integrate PD-1 with TIM-3, LAG-3, TIGIT, 2B4, CD160, and other co-inhibitory receptors, coupled with progressive functional impairment (52, 53). Blackburn and colleagues demonstrated that exhausted CD8^+^ T cells in chronic infection are regulated by the cooperative engagement of several inhibitory pathways, not by a single dominant checkpoint (54). This principle is particularly salient in tumor immunology: a PD-1-expressing T cell in the tumor microenvironment may be activated, dysfunctional, chronically stimulated, or transitioning between states. Consequently, exhaustion is best defined through multimodal evidence, combining phenotype, function, transcriptional state, clonotype, and chromatin accessibility, rather than by inhibitory receptor expression alone (55, 56).

TOX and NR4A-family transcription factors contribute to exhaustion-associated regulatory programs, but neither should be interpreted as a binary marker (57–59). Exhausted populations are heterogeneous: TCF1-positive progenitor-like cells retain self-renewal and can expand after PD-1 blockade, whereas terminally exhausted cells have limited proliferative recovery (60, 61). Thus, reversibility depends on differentiation hierarchy and tumor reactivity, not simply on checkpoint abundance. Exhaustion also has an epigenetic dimension. PD-1 blockade can transiently reinvigorate exhausted T cells, but exhausted cells may retain stable chromatin features that constrain durable memory differentiation (62). Studies of chronic antigen stimulation have shown that exhausted T cells can remain epigenetically scarred even after antigen reduction, limiting their recall capacity and full conversion into conventional memory T cells (63). This concept matters for hematological malignancies because tumor debulking or immunotherapy-induced remission may not fully reset the T cell compartment. A patient who has achieved cytoreduction may still harbor T cells with an exhaustion history that affects subsequent CAR-T cell manufacturing, bispecific antibody responsiveness or immune surveillance (64).

Exhaustion also leaves an epigenetic history. Checkpoint blockade may transiently improve function without fully restoring conventional memory differentiation, and prior chronic stimulation may continue to influence subsequent cellular therapy even after tumor reduction (63–65). In MM bone marrow, checkpoint expression, CD57 positivity, CD28 loss and impaired function coexist, illustrating why multimodal assessment is required (65). Accordingly, exhaustion should be assigned only when phenotype, function and state are concordant. For clinical studies, co-inhibitory receptor co-expression should be interpreted together with proliferative capacity, cytokine production, clonotype behavior and, where feasible, transcriptional or epigenetic features (**Figure 1**).

### 2.2 T cell senescence

T cell senescence is related to, but mechanistically distinct from, exhaustion. It is associated with cumulative cell division, aging, telomere erosion, DNA-damage responses and impaired cell-cycle re-entry (66–68). Senescent-like T cells may retain granzyme or perforin expression yet fail to expand sufficiently for CAR-T cell manufacture or sustained serial killing.

Age reduces naïve T cell output and repertoire diversity while increasing differentiated memory and effector populations (69). Goronzy and Weyand describe T cell aging in terms of impaired self-renewal, dysregulated quiescence, and cellular senescence, emphasizing that aging compromises both the maintenance and responsiveness of the T cell pool (70). This is especially relevant to multiple myeloma, chronic lymphocytic leukemia, and many lymphomas, which arise predominantly in older adults. In these diseases, T cell dysfunction is not solely tumor-induced; it is superimposed on an already aging immune system. This baseline shift is especially relevant in MM, chronic lymphocytic leukemia and many lymphomas, but age alone is an imprecise surrogate: treatment history, chronic infection, disease burden and tissue niche can produce substantial inter-patient variation (71–74).

Practical senescence-associated features include CD27 or CD28 loss, CD57 and KLRG1 expression, shortened telomeres and poor proliferative responses (75–78). None is specific in isolation. CD57-positive or KLRG1-positive cells can be functional late effectors, and the clinical meaning depends on tissue, antigen history and assay context. Functional proliferation and longitudinal persistence are therefore more informative than phenotype alone.

The clinical significance of senescence is particularly evident in autologous cellular therapy. CAR-T cell products are manufactured from a patient’s own T cells, and the quality of this starting population directly shapes the final product (66). A T cell compartment enriched for CD57^+^KLRG1^+^CD28^−^ cells, terminal effector cells, or exhausted populations may yield a product with diminished proliferative reserve and limited persistence (79). CAR design can add a second stress: tonic signaling may induce exhaustion-like changes before antigen encounter, demonstrating that patient-intrinsic cell quality and engineered signaling jointly determine fitness (80). Although these findings pertain to CAR design rather than to patient age, they reinforce a broader principle: both the cell-intrinsic state of the T cell and the imposed signaling architecture jointly determine therapeutic fitness (81) (**Figure 1**).

### 2.3 Terminal differentiation

Terminal differentiation is a developmental end state rather than a synonym for exhaustion. Terminally differentiated or TEMRA-like cells can express GZMB, PRF1, NKG7, CX3CR1 and re-expressed CD45RA, retaining immediate cytotoxicity while showing reduced proliferative reserve, plasticity and persistence (82) (83).

Human terminal effector memory populations are often discussed in relation to TEMRA cells, which re-express CD45RA after antigen experience. David Masopust et al. defines TEMRA cells as a human memory T cell subset that re-expresses CD45RA and displays several characteristics associated with senescence (84). CX3CR1 can also help distinguish cytotoxic effector-like memory CD8^+^ T cell populations from subsets with greater proliferative potential. Böttcher and colleagues showed that CX3CR1 expression classifies memory CD8^+^ T cells with cytotoxic effector function, whereas other memory subsets retain greater proliferative capacity (85, 86). These observations underscore that cytotoxicity and persistence are separable properties. A population can be excellent at immediate killing yet poor at sustaining long-term immune pressure (87).

This distinction is clinically important. Less differentiated central-memory and stem-like memory populations generally support durable adoptive therapy, whereas terminal effectors may provide rapid but short-lived killing (17, 88). During bispecific-antibody treatment, an endogenous pool dominated by terminal or senescent states may respond initially yet fail under repeated engagement. High tumor burden can further accelerate this loss of function (89). Mixed phenotypes are common: a cytotoxic CX3CR1-positive cell may also express inhibitory receptors, and a CD57-positive cell may be senescent-like or simply late differentiated. Classification should therefore integrate transcriptional state, antigen specificity, TCR clonality, proliferation, cytokine production, killing and persistence rather than rely on a small marker panel (90) (**Figure 1**).

### 2.4 T cell fitness as an integrating framework

T cell fitness is an integrating, task-dependent concept. It comprises the measurable properties needed for a specific therapy: cellular availability, memory reserve, proliferative competence, cytotoxicity, metabolic resilience, resistance to chronic stimulation and persistence. Exhaustion, senescence and terminal differentiation reduce different components of this profile. For CAR-T cell therapy, the relevant substrate begins at leukapheresis and must support activation, engineering, expansion and long-term persistence. Bispecific antibodies depend on the quantity, localization and durability of endogenous T cells. Checkpoint blockade requires tumor-reactive cells in a reversible inhibitory state. Consequently, the same patient may have adequate fitness for one platform but not another (91–93). Immune monitoring should therefore move beyond single-marker labeling. Checkpoint receptors, senescence-associated markers, differentiation markers and cytotoxic molecules should be interpreted as domains within a multidimensional profile. The objective is not to assign every cell to one immutable category, but to identify which functional capacity is limiting and whether that capacity is potentially modifiable.

### 2.5 A measurable framework for T cell fitness

No single validated clinical score currently captures T cell fitness across hematological malignancies or treatment platforms. We therefore recommend a domain-based “fitness profile” rather than an unvalidated universal cutoff. At minimum, prospective studies should measure cell availability, memory/differentiation composition, proliferative reserve, dysfunction burden and functional competence before therapy and at prespecified longitudinal time points. For research comparisons, each domain can be standardized within an assay and modeled against a modality-specific endpoint, manufacturing success and *in vivo* expansion for CAR-T cells, serial response and immune attrition for bispecific antibodies, or proliferative reinvigoration for checkpoint blockade. A composite index should only be derived after training, calibration and external validation; until then, domain values should be reported separately to avoid false precision (**Table 1**).

**Table 1:** Proposed multidomain assessment of T cell fitness.

Fitness domain	Practical measures	Modality-specific interpretation	Major limitations
Cell availability	Absolute lymphocyte count; CD3^+^, CD4^+^ and CD8^+^ counts; viable leukapheresis yield	Manufacturability for CAR-T cells; endogenous effector reserve for bispecific antibodies	Counts do not measure function and are affected by recent therapy, infection and sampling time
Memory and differentiation reserve	Naïve, TSCM, TCM, TEM and TEMRA fractions; CD27, CD28, CCR7, CD45RA/RO, TCF1	Expansion and persistence potential; less differentiated states generally favor durable cellular therapy	Marker definitions and tissue distributions vary; phenotype is not equivalent to antigen specificity
Exhaustion/reversibility	Co-expression of PD-1, TIM-3, LAG-3 and TIGIT; TCF1; TOX/NR4A programs; chromatin state when feasible	Distinguishes potentially expandable progenitor-like states from terminal dysfunction	PD-1 alone is nonspecific; transcriptional signatures are platform- and context-dependent
Senescence/replicative reserve	CD57, KLRG1, CD27/CD28 loss; telomere length; DNA-damage markers; cell-cycle re-entry	Identifies limited proliferative reserve relevant to manufacture and repeated engagement	Late differentiation and senescence overlap; telomere assays are not standardized for routine use
Functional competence	Antigen- or CD3/CD28-induced proliferation; cytokine production; degranulation; serial killing	Directly tests expansion and killing capacity required by the intended modality	Assays are labor intensive and results depend on stimulus, target cells and culture conditions
Metabolic resilience	Mitochondrial mass/potential, spare respiratory capacity, glycolytic reserve and stress-response assays	May predict performance during manufacture, high antigen load and chronic stimulation	Mostly research assays; pre-analytic and platform variability are substantial
Clonality and repertoire	TCR diversity, dominant-clone frequency, clonotype persistence and tumor reactivity where available	Assesses repertoire reserve and tracks expansion or attrition of responding clones	Expanded clones may be protective, bystander or dysfunctional; antigen specificity is often unknown
Longitudinal kinetics	CAR transgene copies, blood/tissue expansion, persistence, B-cell aplasia, serial immune phenotype	Provides the most therapy-proximal evidence of fitness after treatment	Post-treatment measures are not available for pretreatment selection and can be confounded by tumor burden

## 3 Disease-specific landscapes of T cell dysfunction in hematological malignancies

**Figure 2:**
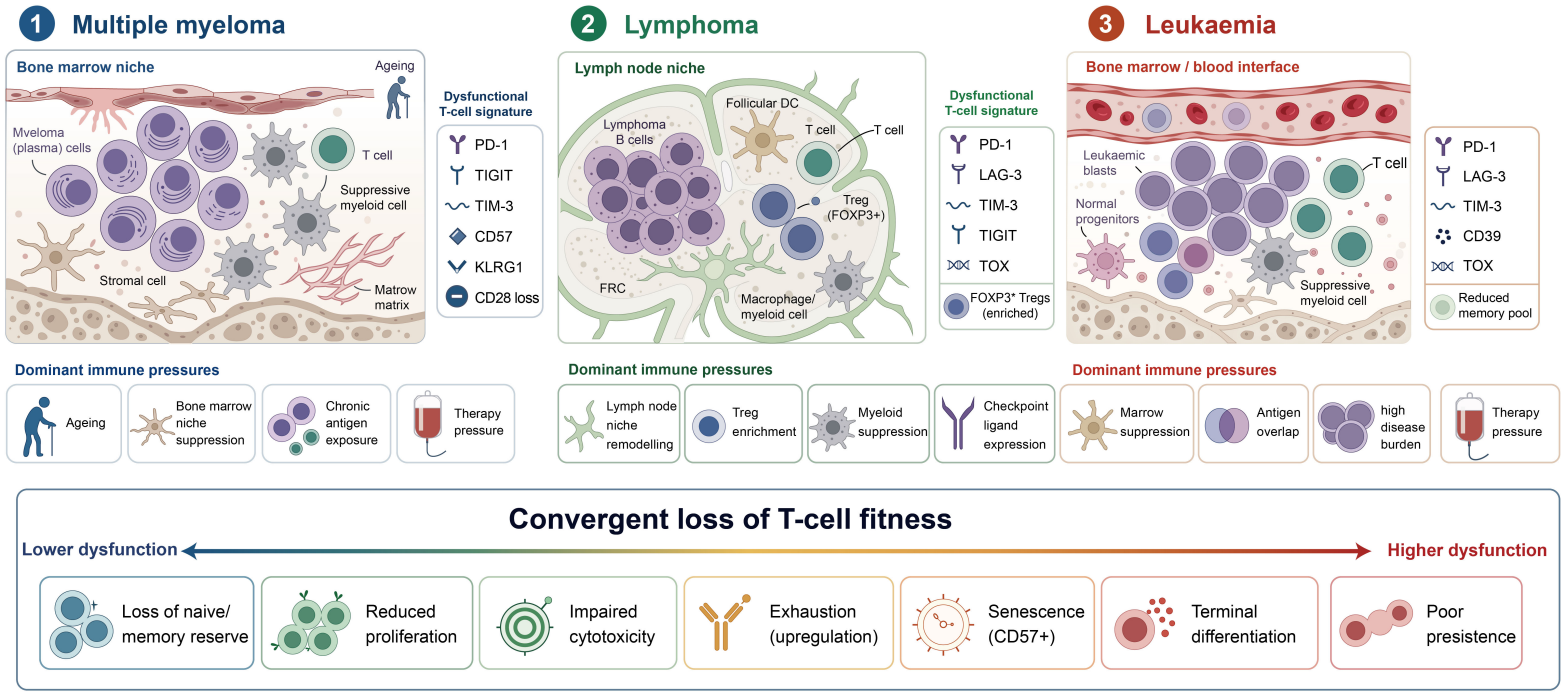
Disease-specific T cell fitness landscapes. MM is shaped by marrow suppression, immune aging and cumulative therapy; lymphoma by lymph-node architecture and suppressive immune contexture; ALL by product persistence and antigen escape; and AML by marrow suppression plus target overlap with normal hematopoiesis. Clinical interpretation: the same biomarker can have different meanings across diseases.

T cell dysfunction varies by malignant lineage, anatomical niche, age distribution, disease burden and treatment history (94–96). The same marker can therefore have different implications across diseases. A disease-specific comparison is more informative than asking whether all blood cancers share a generic exhausted state (**Figure 2**).

### 3.1 Multiple myeloma: ageing, marrow niche suppression and immune redirection

Multiple myeloma provides perhaps the clearest model in which ageing-associated and therapy-induced T cell dysfunction intersect. Epidemiologically, myeloma is a disease of older adults: the American Cancer Society reports that most patients are at least 65 years old at diagnosis and that the average age at diagnosis is approximately 69 years (97). This age distribution matters immunologically. The T cell compartment of many patients is already shaped by immunosenescence before exposure to multiple lines of therapy (98). At the same time, myeloma arises and persists in the bone marrow, an immune-regulatory niche in which malignant plasma cells interact with stromal cells, myeloid cells, osteoclast-lineage cells, cytokine networks and extracellular matrix components (99). Thus, the T cell state in myeloma is not simply a peripheral immune phenotype; it is conditioned locally by the marrow microenvironment (100) (**Figure 2**). MM studies have identified checkpoint co-expression, CD57 positivity, CD28 loss, reduced proliferation and impaired cytokine or degranulation responses (65, 101). The strength of this evidence is its functional and tissue-specific characterization; its limitation is that relatively small cohorts and heterogeneous prior therapy make it difficult to assign causality to disease, age or treatment separately.

Single-cell studies further show that MM is not uniformly dominated by canonical exhaustion. Large checkpoint-expressing clones can accumulate during disease evolution and associate with treatment resistance, whereas other datasets emphasize antigen-driven terminal-memory differentiation rather than a classic exhaustion program (33, 102). These apparently conflicting findings are compatible with a heterogeneous fitness landscape and caution against using one signature across all patients.

This heterogeneity is clinically relevant because BCMA-directed CAR-T cells and bispecific antibodies are often delivered after extensive therapy. Response may be limited by different mechanisms: poor starting-cell quality and persistence for CAR-T cells, or progressive attrition of the endogenous T cell pool during repeated bispecific-antibody exposure. Infection and immune-reconstitution outcomes further indicate that antitumor response alone is not a complete measure of therapeutic fitness.

### 3.2 B-cell lymphoma: lymph node architecture and suppressive immune contexture

B-cell lymphomas represent a different immune problem. Unlike myeloma, which is embedded in the bone marrow plasma-cell niche, many lymphomas grow within lymphoid tissues that are anatomically designed to organize T cell-B-cell interactions. The lymph node provides chemokine gradients, follicular dendritic cell networks, stromal reticular structures and specialized T cell zones. Malignant B cells can exploit this architecture to create a suppressive or spatially distorted immune environment (103). In this context, T cell dysfunction is shaped by chronic antigen stimulation, tumor-associated macrophages, regulatory T cells, checkpoint ligand expression, prior chemoimmunotherapy and disruption of normal lymphoid organization (104).

The success of CD19-directed CAR-T cell therapy in aggressive B-cell lymphomas established lymphoma as a major immunotherapy success story, but also revealed the importance of T cell fitness. In the pivotal ZUMA-1 study, axicabtagene ciloleucel produced high response rates in refractory large B-cell lymphoma; long-term follow-up showed that a subset of patients achieved sustained responses, but many patients still relapsed or failed to achieve durable remission (105, 106). Mechanistically, lymphoma CAR-T failure is not explained solely by antigen loss. It can occur through inadequate CAR-T expansion, poor persistence, intrinsic dysfunction of the infusion product, suppressive tumor microenvironment features or failure to generate broader endogenous anti-tumor immunity (15).

Several studies illustrate this point. An analysis integrating clinical CAR-T pharmacology and pre-infusion transcriptomes found that CAR-T expansion and persistence vary widely among patients and are linked to clinical response; non-durable responses were associated with deficits in proliferative and functional capacity characteristic of exhaustion and terminal differentiation (107). Another single-cell study of large B-cell lymphoma treated with CD19 CAR-T cells showed that expansion of proliferative memory-like CD8 clones was a hallmark of response to tisagenlecleucel, whereas nonresponse to axicabtagene ciloleucel was associated with CAR-T regulatory populations that could suppress conventional CAR-T expansion and drive late relapse in models (108). These findings support a key point: in lymphoma, T cell dysfunction is both cell-intrinsic and niche-dependent. The infused CAR-T product must retain proliferative potential, but it must also function within a tumor site enriched for regulatory and myeloid suppressive signals.

Pretreatment immune contexture, including T cell infiltration and immune gene signatures, has been associated with response and survival after axi-cel, while post-treatment tissue exhaustion has correlated inversely with circulating CAR-T cell levels (109). These observations are clinically relevant but remain largely associative. They support tissue-aware biomarkers in lymphoma rather than direct extrapolation from MM marrow or peripheral blood (**Figure 2**).

### 3.3 Acute lymphoblastic leukemia: potent CAR-T activity, antigen escape and persistence failure

B-cell acute lymphoblastic leukemia (B-ALL) has produced some of the most striking examples of CAR-T cell efficacy, yet it also illustrates how tumor escape and T cell fitness failure intersect (110). CD19-directed CAR-T cells induce deep remissions in children and young adults with relapsed or refractory disease; the initial CTL019 experience showed complete remission in 27 of 30 patients, and the global ELIANA trial subsequently established tisagenlecleucel as a transformative therapy with high response rates and detectable CAR-T persistence in a subset of patients (111).

Nonetheless, relapse after CD19 CAR-T therapy reveals two distinct failure modes: CD19-negative escape, driven by antigen loss or lineage switch, and CD19-positive relapse, reflecting inadequate CAR-T persistence or potency. Orlando and colleagues identified genetic mechanisms of antigen loss, including CD19 mutations and loss of heterozygosity, that eliminate surface antigen (112), while Jacoby and colleagues demonstrated that CAR-mediated immune pressure can trigger lineage switch from B-precursor ALL to a myeloid phenotype, exposing inherent leukemic plasticity (113). These tumor-intrinsic resistance mechanisms are shaped by T cell fitness: robust CAR-T persistence favors selection of antigen-negative variants, whereas weak persistence permits CD19-positive regrowth.

In ALL, the dominant fitness questions are product composition, early expansion and long-term persistence rather than baseline immunosenescence. Rare stem-like memory clones can contribute disproportionately to durable persistence (114), while relapse may be CD19-positive because of insufficient CAR-T cell persistence or CD19-negative because of antigen loss or lineage switch. Biomarkers should therefore combine memory composition and cellular kinetics with antigen status at relapse (**Figure 2**).

### 3.4 Acute myeloid leukemia: marrow suppression, antigen overlap and myeloid immune evasion

Acute myeloid leukemia (AML) represents the most challenging setting for T cell immunotherapy among the hematological malignancies discussed here. AML arises from hematopoietic progenitors and occupies the same marrow niches that sustain normal hematopoiesis, creating two fundamental obstacles (115). First, the bone marrow microenvironment is profoundly remodeled by leukemic blasts, inflammatory cytokines, suppressive myeloid cells, and disrupted stromal interactions. Second, most candidate AML antigens are shared with normal myeloid progenitors or hematopoietic stem and progenitor cells, leaving a narrow therapeutic window for CAR-T cells and other T cell-engaging modalities (116, 117).

The immune landscape of AML is shaped by marrow inflammation and myeloid-driven suppression. As highlighted recently, inflammatory remodeling of the bone marrow niche compromises both malignant and normal hematopoiesis, and leukemias extensively reshape the immune microenvironment, retaining immature immune features that contribute to immunotherapy resistance (118, 119). These properties help explain why checkpoint blockade and CAR-T cell therapy have proven less effective in AML than in B-cell malignancies (120).

In AML, exhaustion can affect early memory compartments after allogeneic transplantation, but the therapeutic problem extends beyond T cell state. Suppressive myeloid niches and shared expression of targets such as CD33 or CD123 on normal progenitors constrain the safety window (121, 122). Restoring T cell function alone cannot overcome an unfavorable target landscape; fitness and antigen selectivity must be evaluated together (**Figure 2**).

### 3.5 Integrating disease context into T cell fitness

Across diseases, the limiting fitness dimension differs. MM emphasizes marrow suppression, immune aging and cumulative therapy; lymphoma emphasizes tissue immune contexture and product-niche interaction; ALL emphasizes memory composition, persistence and antigen escape; and AML combines marrow immune dysfunction with target overlap on normal hematopoiesis.

Accordingly, PD-1, CD57 or KLRG1 cannot be assigned a uniform meaning. Their interpretation depends on disease, tissue, co-expressed markers, function, clonality and treatment timing. **Table 2** summarizes these disease-specific differences and the principal evidence gaps. The practical consequence is that biomarker panels and therapeutic thresholds should be developed separately for each modality and disease. A marker that predicts CAR-T cell manufacturing failure may not predict response to a bispecific antibody, and a blood phenotype may not reflect a marrow or lymph-node compartment (**Figure 2**).

**Table 2:** Disease-specific determinants and consequences of reduced T cell fitness.

Disease context	Dominant fitness pressures	Therapy-specific consequences
Multiple myeloma	Older age distribution; marrow stromal/myeloid suppression; chronic antigen; extensive prior therapy; hypogammaglobulinemia	Reduced autologous product quality or persistence; endogenous T cell attrition during prolonged bispecific-antibody exposure; infection vulnerability
B-cell lymphoma	Disrupted lymph-node architecture; Tregs; macrophages; checkpoint-rich niches; prior chemoimmunotherapy	CAR-T cell trafficking, expansion and persistence depend on both product state and tissue contexture
B-cell ALL	High antigen pressure; treatment-associated lymphodepletion; lineage plasticity; variable product persistence	CD19-positive relapse suggests inadequate persistence; CD19-negative relapse suggests antigen escape or lineage switch
AML	Suppressive myeloid marrow niche; inflammatory remodeling; post-transplant exhaustion; target overlap with normal progenitors	Limited efficacy from both T cell dysfunction and a narrow antigen-safety window

ALL, acute lymphoblastic leukemia; AML, acute myeloid leukemia; MM, multiple myeloma; TCM, central-memory T cell; TSCM, stem-cell-memory T cell; Treg, regulatory T cell.

## 4 Impact of T cell ageing and exhaustion on immunotherapy

T cell-directed therapies interrogate different components of the fitness profile. CAR-T cell therapy requires a viable starting population, successful manufacture, *in vivo* expansion and persistence. Bispecific antibodies repeatedly recruit endogenous T cells. Checkpoint blockade requires tumor-reactive cells whose inhibitory state remains reversible. Aging, exhaustion, senescence and terminal differentiation therefore impair distinct steps rather than producing a uniform treatment effect.

### 4.1 CAR-T cell therapy

CAR-T cell therapy offers the clearest illustration of why baseline T cell fitness is decisive. Autologous CAR-T cell products are manufactured from a patient’s own T cells, typically after multiple lines of chemotherapy, corticosteroids, immunomodulatory agents, antibody-based therapies, transplantation or bridging treatment (123). The starting leukapheresis product is therefore far from immunologically neutral; it carries the cumulative imprint of age, tumor burden, chronic antigen exposure and prior therapy (124). If the collected T cells are enriched for terminally differentiated, senescent or exhausted populations, they may transduce and expand poorly, acquire dysfunction during manufacturing, or fail to persist after infusion. Conversely, products enriched for less differentiated, memory-like cells are more likely to expand robustly and sustain antitumor pressure (125).

Strong clinical evidence supports this principle. In a landmark study of CD19 CAR-T cells in chronic lymphocytic leukemia, Fraietta and colleagues showed that complete responders possessed CAR-T cell products enriched for memory-related gene programmes, including IL-6–STAT3 signatures, whereas non-responders exhibited upregulation of effector differentiation, glycolysis, exhaustion and apoptosis pathways. Sustained remission was associated with a higher frequency of CD27^+^CD45RO^-^CD8^+^ T cells prior to CAR-T cell generation, demonstrating that pre-manufacturing T cell state can influence clinical outcome. This finding extends beyond CLL: it reframes CAR-T cell efficacy as a function not only of the engineered receptor but also of the cellular substrate into which that receptor is introduced (17).

The importance of memory-like T cells is further reinforced by clonal tracking studies. Biasco and colleagues used integration-site analysis to show that T memory stem cell clones, though rare in the infused product, contributed substantially to early antileukemic responses and long-term CAR-T cell persistence (114). More broadly, adoptive T cell therapy studies consistently demonstrate that persistence is governed by differentiation state: less differentiated T cells retain greater proliferative potential, metabolic flexibility and self-renewal capacity than terminal effector cells (126). These observations provide a biological rationale for manufacturing strategies aimed at enriching stem-like memory or central memory populations, limiting excessive activation during culture, or employing cytokine conditions that preserve less differentiated states.

Prior therapy can compromise CAR-T cell fitness through several mechanisms. Alkylating agents, purine analogues, corticosteroids and repeated lymphotoxic regimens may deplete T cell numbers, narrow T cell receptor diversity, drive terminal differentiation or erode proliferative reserve (127, 128). In multiple myeloma, patients frequently receive CAR-T cells late in the disease course, after exposure to proteasome inhibitors, immunomodulatory drugs, anti-CD38 antibodies, corticosteroids and sometimes transplantation. In lymphoma and leukaemia, prior chemotherapy and bridging therapy can similarly reshape lymphocyte composition before leukapheresis (129). These pressures do more than reduce absolute lymphocyte counts; they can fundamentally alter the composition of the T cell pool from which the final CAR-T cell product is derived. Thus, fitness prior to leukapheresis is a clinically relevant variable, not merely a correlative biomarker (130).

After infusion, CAR-T cells encounter a second layer of fitness selection. They must proliferate *in vivo*, traffic to tumor sites, form productive immune synapses, kill malignant cells and persist long enough to prevent relapse. CAR design directly modulates this process through costimulatory domains, which provide distinct signaling programmes: CD28-containing CARs are typically associated with rapid activation and expansion, whereas 4-1BB-containing CARs are often linked to enhanced persistence and mitochondrial fitness (131). Mechanistically, Long and colleagues demonstrated that tonic CAR signalling can drive early CAR-T cell exhaustion through antigen-independent CAR clustering, and that CD28 co-stimulation can augment, whereas 4-1BB co-stimulation can mitigate, exhaustion induced by persistent CAR signalling (80). These data do not imply universal superiority of one costimulatory domain across all contexts, but they establish that CAR architecture shapes the balance between activation, exhaustion and persistence.

**Figure 3:**
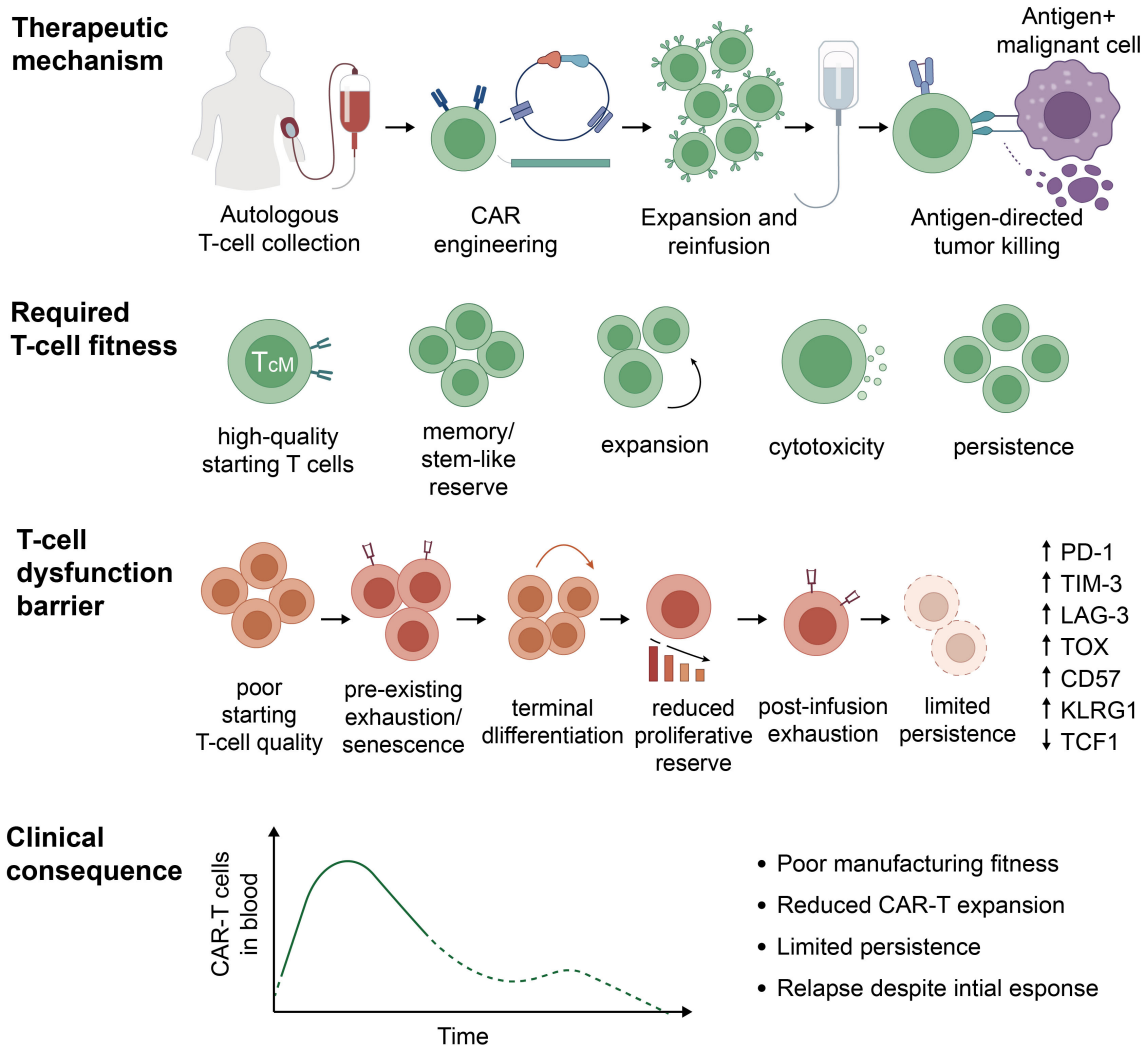
CAR-T cell therapy: therapeutic mechanism, T cell fitness requirements, dysfunction barriers and clinical consequences. The autologous chimeric antigen receptor (CAR)-T workflow involves T cell collection, CAR engineering, ex vivo expansion and patient reinfusion to mediate antigen-directed tumor killing. Durable efficacy depends on high-quality starting T cells (e.g. central memory T cells), a stem-like memory reservoir, robust proliferative expansion, cytotoxic function and long-term *in vivo* persistence. Pre-existing T cell dysfunction, including poor initial quality, senescence, terminal differentiation and exhaustion, constrains proliferative reserve and post-infusion persistence, resulting in poor manufacturing fitness, blunted CAR-T expansion, limited durability and disease relapse despite initial responses. CAR, chimeric antigen receptor; TCM, central memory T cell; PD-1, programmed cell death protein 1; TIM-3, T cell immunoglobulin and mucin-domain containing-3; LAG-3, lymphocyte-activation gene 3; TOX, thymocyte selection-associated high mobility group box protein; CD57, cluster of differentiation 57; KLRG1, killer cell lectin-like receptor G1; TCF1, T cell factor 1.

After infusion, high antigen burden, repeated encounter, tonic signaling, inflammatory stress, metabolic insufficiency and suppressive niches can accelerate CAR-T cell attrition (132–134). Antigen-negative relapse and antigen-positive relapse should be separated: the former indicates tumor escape, whereas the latter more strongly implicates inadequate expansion, persistence or function. Real-world interpretation is complicated by variation in disease burden, bridging therapy, product, toxicity management and post-infusion infection; these factors should be incorporated into biomarker models rather than treated as noise (**Figure 3**).

### 4.2 Bispecific antibodies

Bispecific antibodies create a different therapeutic challenge because they do not require ex vivo manufacture (135). Instead, they recruit the patient’s endogenous T cells *in vivo*, typically by binding CD3 on T cells and a tumor-associated antigen on malignant cells. In multiple myeloma, approved and emerging bispecific antibodies target antigens such as BCMA and GPRC5D; in B-cell malignancies, CD19×CD3 and CD20×CD3 formats have also been developed (15). The apparent practical advantage of bispecific antibodies, off-the-shelf administration, is also their biological vulnerability: efficacy depends on the availability, localization and repeated functional engagement of endogenous T cells that may already be exhausted, senescent or numerically depleted (136).

Clinical trials in relapsed or refractory myeloma illustrate both the power and the limitations of this approach. In the phase I–II MajesTEC-1 study, teclistamab, a BCMA×CD3 bispecific antibody, was tested in patients with relapsed or refractory myeloma after at least three previous lines of therapy, including triple-class exposure; among 165 treated patients, 77.6% had triple-class refractory disease and the median number of prior therapy lines was five. These eligibility and baseline features matter for interpretation (7). The endogenous T cells being recruited by teclistamab were not naïve immune effectors in an untreated host; they came from heavily pretreated patients with advanced myeloma and extensive prior immune perturbation.

Single-cell and functional studies have begun to define why some patients respond to bispecific antibodies whereas others do not. Friedrich et al. reported that the pre-existing T cell landscape influences response to BCMA-targeted T cell engagers in myeloma; clonal expansion of effector CD8^+^ T cells was associated with therapeutic activity, whereas an abundance of exhausted CD8^+^ clones predicted response failure (89). Verkleij et al. similarly showed that T cell characteristics influence response and resistance to teclistamab and talquetamab in myeloma, using ex vivo killing assays and immune profiling of patient samples (137). These findings support a key mechanistic distinction from CAR-T cells: for bispecific antibodies, the limiting substrate is not the manufactured product but the patient’s residual endogenous T cell ecosystem.

Continuous stimulation is a particular concern for bispecific antibodies. Unlike a single CAR-T cell infusion, many bispecific antibodies are administered repeatedly or continuously until progression or intolerance (16). Persistent CD3 engagement may drive progressive exhaustion, reduced cytokine production, impaired serial killing and T cell attrition. A study modelling continuous exposure to CD19×CD3 bispecific molecules showed that continuous treatment induced T cell exhaustion and declining function, whereas treatment-free intervals promoted transcriptional reprogramming and functional reinvigoration (138). Although this study was not limited to myeloma, it provides a mechanistic rationale for investigating fixed-duration dosing, intermittent schedules or response-adapted treatment breaks in T cell-engager therapy.

Infections during bispecific-antibody treatment are multifactorial and cannot be attributed to T cell exhaustion alone. Plasma-cell depletion, hypogammaglobulinemia, neutropenia, lymphopenia, prior therapy and cumulative exposure all contribute (139, 140). Real-world cohorts extend pivotal-trial observations to older and more heterogeneous patients, but treatment schedules, prophylaxis and follow-up differ. Consequently, infection outcomes should be interpreted alongside cellular fitness and humoral immune reconstitution. Treatment duration and sequencing remain unresolved. Continuous exposure may promote chronic stimulation, and less frequent or finite-duration approaches may reduce infection and permit partial functional recovery, but current evidence is not sufficient to define a universal stopping rule (141). Prospective trials should integrate serial T cell phenotype and function with tumor response, minimal residual disease and infection outcomes.

**Figure 4:**
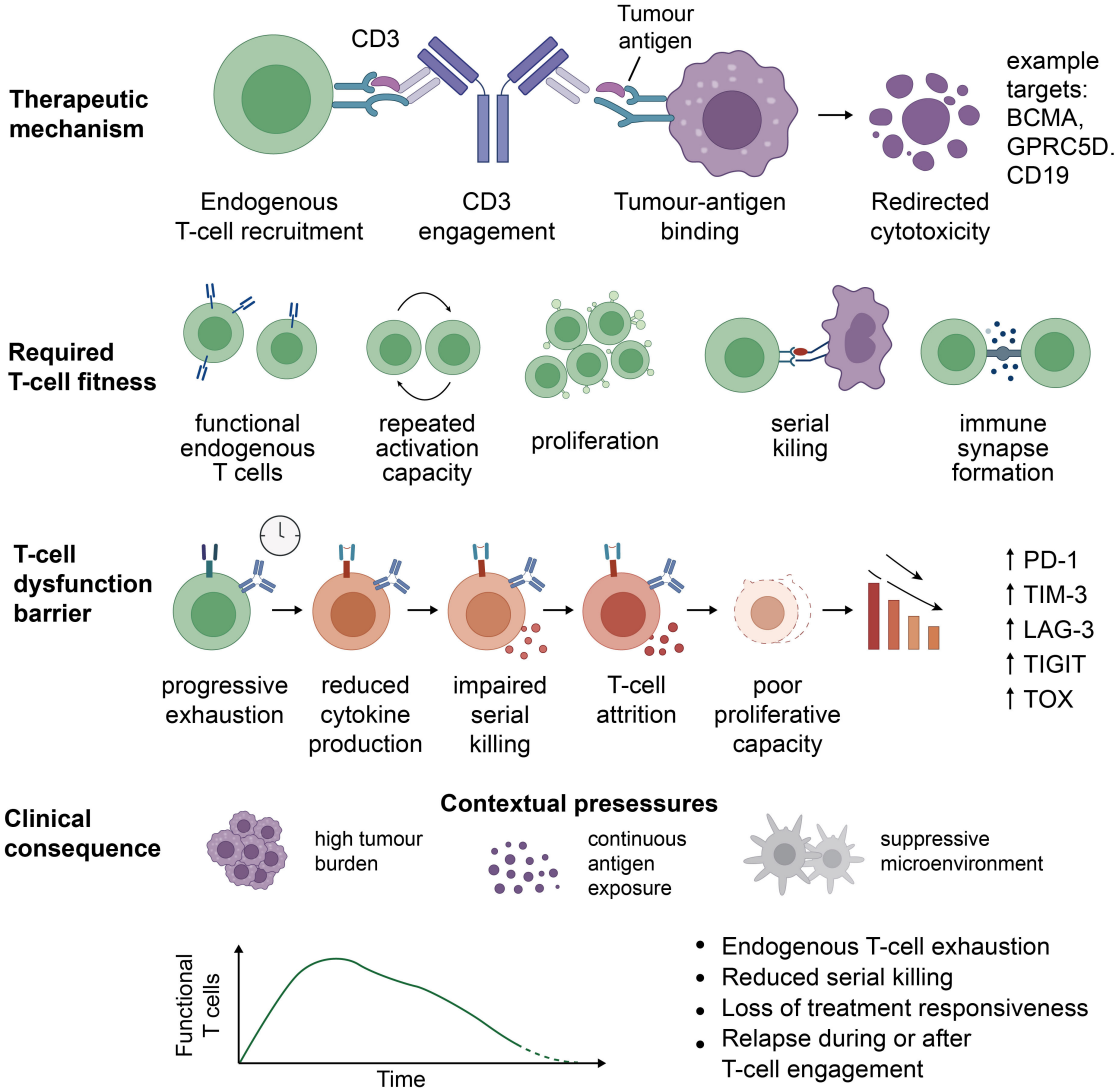
T cell-engaging bispecific antibody therapy: mechanism of action, T cell fitness prerequisites, dysfunction barriers and contextual clinical pressures. Bispecific antibodies co-engage CD3 on endogenous T cells and tumor surface antigens (exemplified by BCMA, GPRC5D and CD19) to redirect T cell cytotoxicity against malignant cells. Sustained anti-tumor activity requires functional endogenous T cells with capacity for repeated activation, proliferation, serial killing and stable immune synapse formation. Progressive T cell exhaustion, exacerbated by high tumor burden, continuous antigen exposure and an immunosuppressive microenvironment, reduces cytokine production, impairs serial killing, drives T cell attrition and abrogates proliferative capacity, leading to endogenous T cell exhaustion, diminished tumor killing, lost treatment responsiveness and on-treatment or post-treatment relapse. CD3, cluster of differentiation 3; BCMA, B-cell maturation antigen; GPRC5D, G protein-coupled receptor class C group 5 member D; CD19, cluster of differentiation 19; PD-1, programmed cell death protein 1; TIM-3, T cell immunoglobulin and mucin-domain containing-3; LAG-3, lymphocyte-activation gene 3; TIGIT, T cell immunoreceptor with Ig and ITIM domains; TOX, thymocyte selection-associated high mobility group box protein.

Sequencing with CAR-T cells remains unresolved. Prior bispecific antibody exposure might deplete or exhaust endogenous T cells and potentially affect later T cell-redirection strategies. Conversely, CAR-T cell relapse may leave behind an immune landscape in which bispecific antibodies retain activity, especially when targeting a different antigen. Current data are still evolving, and no single sequence is universally established across disease types, targets and patient populations. The conceptual implication is that sequencing should eventually incorporate immune fitness, not only antigen expression or prior response (**Figure 4**).

### 4.3 Checkpoint blockade

Checkpoint blockade provides a third and fundamentally distinct example of T−cell dysfunction in therapy. In classical Hodgkin lymphoma, PD−1 blockade has been highly active, largely because Reed–Sternberg cells frequently exploit PD−1 ligand biology through 9p24.1 alterations and inflammatory immune niches (142). The FDA granted accelerated approval to nivolumab for classical Hodgkin lymphoma after autologous transplantation and brentuximab vedotin failure; the approval summary reported an objective response rate of 65% in 95 patients in the evaluated population (143). This success demonstrates that checkpoint−dependent immune suppression can be therapeutically actionable in selected hematological malignancies.

However, checkpoint blockade has not translated uniformly across blood cancers. Multiple myeloma is the clearest cautionary example. PD−1 expression on T cells in myeloma does not necessarily identify a reversibly exhausted, tumor−reactive compartment that can be safely and effectively rescued. The FDA placed KEYNOTE−183 and KEYNOTE−185 on hold after interim analyses showed an increased risk of death when pembrolizumab was combined with immunomodulatory agents in myeloma. The KEYNOTE−183 concluded that pembrolizumab plus pomalidomide and dexamethasone had an unfavorable benefit–risk profile in relapsed or refractory myeloma; the KEYNOTE−185 study similarly assessed pembrolizumab plus lenalidomide and dexamethasone in treatment−naive, transplant−ineligible myeloma and was stopped after an FDA−requested interim safety evaluation (144, 145).

The myeloma checkpoint experience carries a broader lesson: inhibitory receptor expression is not synonymous with reversible exhaustion. PD−1 may mark activation, chronic stimulation, terminal differentiation, tissue adaptation or dysfunctional states that cannot be productively rescued by PD−1 blockade alone (146). If the dominant problem is senescence, telomere−associated proliferative arrest, loss of memory reserve or terminal differentiation, checkpoint blockade may have limited capacity to restore durable anti−tumor immunity (147). Similarly, if resistance is driven by suppressive myeloid cells, regulatory T cells, defects in antigen presentation or tumor−intrinsic immune escape, PD−1 blockade alone may be insufficient (148).

**Figure 5:**
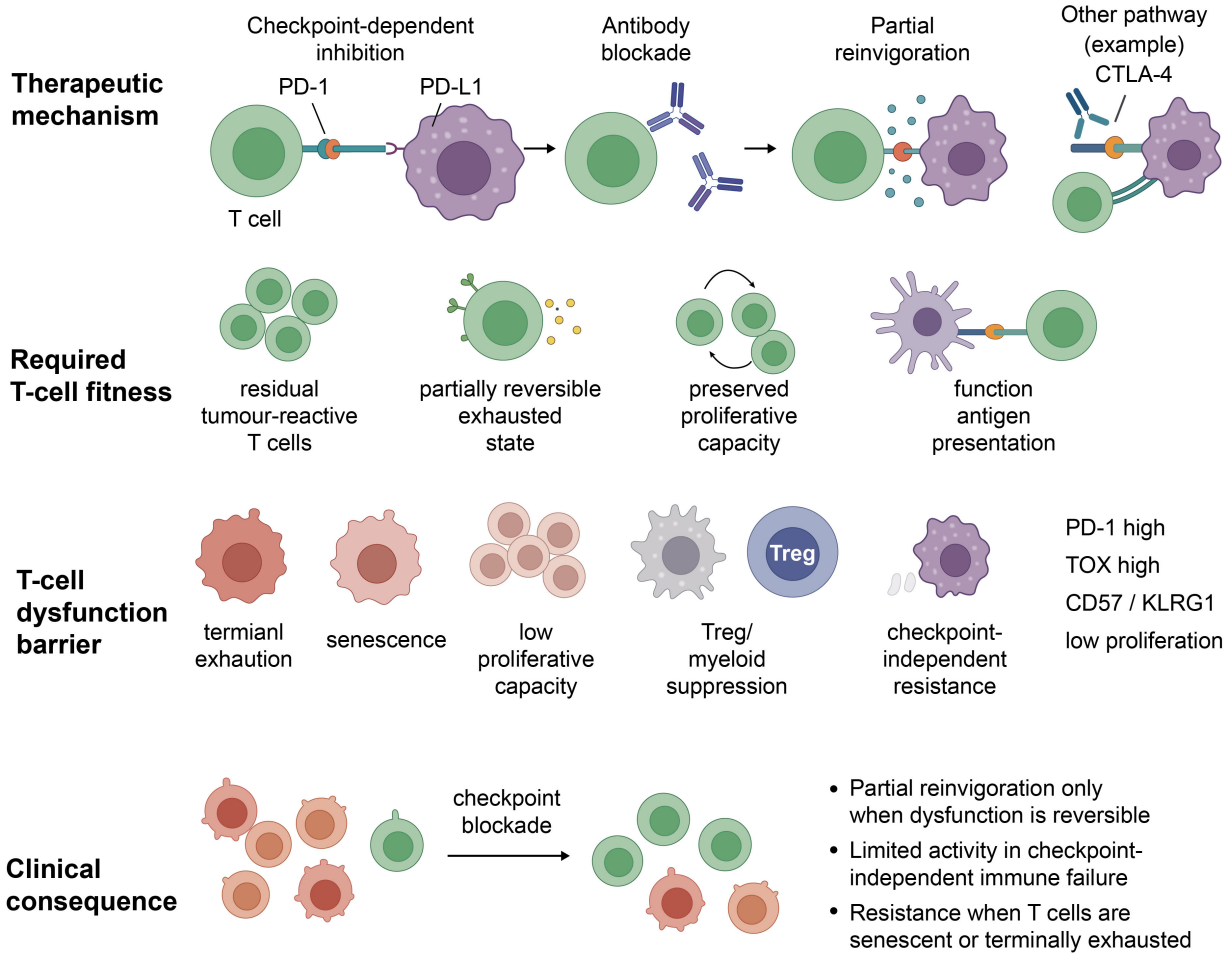
Immune checkpoint blockade therapy: mechanism of action, T cell fitness requirements, resistance barriers and clinical consequences. PD-1/PD-L1 checkpoint blockade relieves inhibitory signalling to partially reinvigorate tumor-reactive T cells; additional pathways such as CTLA-4 blockade can further augment anti-tumor immunity. Therapeutic efficacy relies on residual tumor-reactive T cells in a partially reversible exhausted state, preserved proliferative capacity and competent antigen presentation. Terminal T cell exhaustion, senescence, impaired proliferation, immunosuppressive cell populations (regulatory T cells, myeloid cells) and checkpoint-independent resistance represent key therapeutic barriers. Checkpoint blockade achieves only partial reinvigoration of reversibly dysfunctional T cells, with limited activity against terminally exhausted or senescent T cells and checkpoint-refractory immune failure. PD-1, programmed cell death protein 1; PD-L1, programmed death-ligand 1; CTLA-4, cytotoxic T-lymphocyte-associated protein 4; Treg, regulatory T cell; TOX, thymocyte selection-associated high mobility group box protein; CD57, cluster of differentiation 57; KLRG1, killer cell lectin-like receptor G1.

This does not diminish the importance of checkpoint biology. Rather, it argues for a more precise model. Checkpoint blockade is most likely to be effective when tumor−reactive T cells remain present, when dysfunction is at least partially reversible, and when inhibitory receptor signalling is a dominant mechanism of suppression. By contrast, CAR-T cells can bypass aspects of endogenous antigen recognition but require manufacturable and persistent T cells. Bispecific antibodies can redirect polyclonal endogenous T cells but may intensify chronic stimulation and exhaustion. Therefore, each immunotherapy probes a different dimension of T−cell fitness (**Figure 5**).

### 4.4 Convergent and distinct mechanisms of failure

Different T cell-directed therapies fail through partially distinct mechanisms (**Figures 3**–**5**). CAR-T cell failure may reflect poor starting-cell quality, manufacturing limitations, inadequate expansion, limited persistence, chronic signaling or antigen escape. Bispecific-antibody failure more often reflects limitations of the endogenous T cell pool, tumor burden, continuous CD3 engagement and progressive attrition. Checkpoint blockade fails when tumor-reactive T cells are absent, dysfunction is terminal, or checkpoint-independent mechanisms dominate (149). This distinction should determine what is measured. CAR-T cell studies should assess leukapheresis yield and phenotype, manufacturing performance, product composition, expansion and persistence. Bispecific-antibody studies should assess endogenous T cell abundance, function, clonality, disease burden, schedule and immune reconstitution. Checkpoint studies should assess tumor reactivity, antigen presentation, ligand biology and progenitor-like versus terminal exhaustion. None of these domains has a universally validated cutoff.

## 5 Restoring T cell fitness: future therapeutic strategies

**Figure 6:**
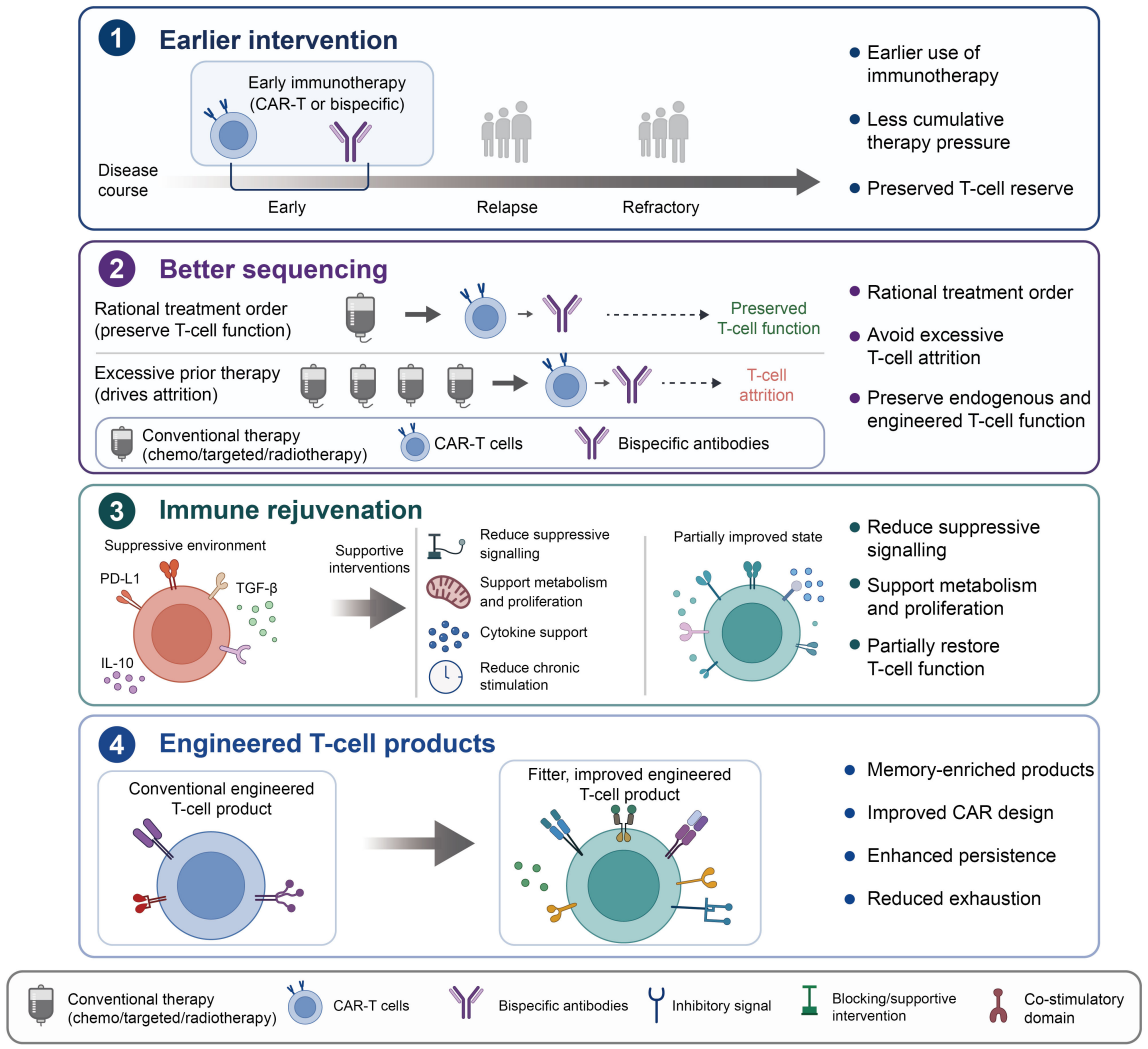
Strategies to preserve or restore T cell fitness. The framework includes four complementary approaches: earlier collection and treatment before cumulative attrition; rational bridging, sequencing and duration; selective immune or metabolic rejuvenation; and engineering of memory-enriched, exhaustion-resistant or alternative allogeneic/CAR-NK products. Clinical interpretation: preservation of a usable immune substrate should be considered alongside tumor cytoreduction and antigen selection. CAR, chimeric antigen receptor; NK, natural killer.

Strategies to improve T cell-directed immunotherapy should be organized around prevention, selective restoration and engineering. The objective is not to indiscriminately increase activation or cytokine release, but to preserve the specific properties—memory, proliferation, serial killing and persistence—needed for the chosen modality (**Figure 6**).

### 5.1 Preserving T cell fitness before it is lost

One obvious strategy is to deploy T cell-redirecting therapies earlier in the disease course, before the T cell compartment has been extensively damaged by repeated relapse, chronic antigen exposure, corticosteroids, lymphotoxic chemotherapy, or persistent marrow and lymph node suppression. This concept is supported clinically by the movement of CAR-T cell therapy into earlier relapsed settings. In multiple myeloma, the FDA approval summary for idecabtagene vicleucel after two or more prior lines was based on KarMMa-3, in which ide-cel significantly prolonged progression-free survival versus standard regimens, with a median progression-free survival of 13.3 months versus 4.4 months and a hazard ratio of 0.49 (150). Similarly, CARTITUDE-4 evaluated ciltacabtagene autoleucel in lenalidomide-refractory myeloma after one to three previous lines and reported significantly improved progression-free survival versus standard care. These trials were designed primarily around clinical efficacy, but they also support an immunological premise: earlier use may capture T cells before more profound attrition, terminal differentiation and clonal restriction occur (151).

For autologous CAR-T cell therapy, the period before leukapheresis is clinically important. Where disease control permits, collection before repeated lymphotoxic treatment or prolonged high-dose corticosteroid exposure may preserve cell number and function (152, 153). Bridging therapy should be selected primarily to control disease and reduce immediate clinical risk while minimizing additional lymphotoxicity and allowing the required washout. It should not be used with the deliberate aim of maximizing tumor-antigen release or systemic inflammation. High disease burden and inflammatory activation can increase toxicity and chronic stimulation, whereas effective cytoreduction may improve the conditions for CAR-T cell expansion. Local radiotherapy or systemic bridging can be appropriate in selected patients, but choice should be individualized according to disease distribution, kinetics, cytopenias, infection risk and the planned product (130, 140, 162, 163) (**Figure 6**).

### 5.2 Rejuvenating dysfunctional T cells

A second strategy is to rejuvenate T cells that are already dysfunctional. This area holds promise but must be framed with caution. Exhaustion, senescence and terminal differentiation are not equally reversible. Progenitor-like exhausted T cells can respond to checkpoint or cytokine-based stimulation, whereas terminally exhausted or senescent T cells may have fixed epigenetic, proliferative or metabolic constraints (154). Thus, immune rejuvenation should be viewed as partial restoration of specific fitness dimensions rather than complete reversal of dysfunction.

Cytokine, metabolic and epigenetic approaches aim to restore selected fitness dimensions. IL-7 and IL-15 can support survival and memory maintenance, and IL-15-based culture has preserved stem-like features in preclinical CAR-T cell models (155, 156). Mitochondrial support, restrained mTOR signaling and metabolic reprogramming may improve persistence under stress (157). Transient rest can reverse selected exhaustion-associated features in experimental CAR-T cells, but terminal exhaustion and senescence are less likely to be fully reversible (158). These approaches remain largely preclinical and require careful assessment of inflammation, off-target effects and lineage stability.

The tumor microenvironment is an equally important target. In myeloma, suppressive bone marrow stromal and myeloid networks, chronic antigen exposure and high tumor burden can accelerate T cell dysfunction. In lymphoma, lymph node architecture, regulatory T cells and macrophages can limit CAR-T cell function. In bispecific antibody therapy, pre-existing exhausted-like CD8^+^ clones predicted response failure in myeloma, supporting immune monitoring and potential conditioning of the immune repertoire before T cell engagement (89). Rejuvenation therefore should not be restricted to the T cell itself; it should include debulking, myeloid modulation, regulatory T cell reduction and reversal of suppressive marrow or lymph node niches, where supported by disease context and safety (**Figure 6**).

### 5.3 Engineering fitter T cell products

Fitness can also be engineered into the product. Enrichment for stem-like or central-memory populations, shorter or less activating culture, optimized cytokine conditions and construct-specific control of tonic signaling can preserve proliferative reserve. Product design should be evaluated against the intended disease context rather than by a universal assumption that one costimulatory domain or manufacturing method is superior.

Costimulatory domain design is a key example. Long and colleagues showed that antigen-independent tonic CAR signalling can induce early exhaustion and limit anti-tumor efficacy; importantly, in their experimental system, CD28 co-stimulation augmented exhaustion, whereas 4-1BB co-stimulation reduced exhaustion induced by persistent CAR signalling. This does not imply that 4-1BB is universally superior to CD28, as optimal design depends on the antigen, disease context, construct and desired kinetics (80). However, it establishes that receptor architecture can tune exhaustion, persistence and metabolic state.

Gene-edited and armored CAR-T cell products can disrupt exhaustion-associated pathways, resist suppressive ligands or provide controlled cytokine support, although most strategies remain preclinical or early clinical (159) (160) (**Figure 4**). CAR-NK cells provide an adjacent approach that may reduce dependence on heavily pretreated autologous T cells and facilitate off-the-shelf manufacturing; early studies are encouraging, but limited persistence, trafficking and suppressive microenvironments remain substantial barriers. Metabolic engineering, including mitochondrial support, nutrient-sensing modification and controlled cytokine signaling—should likewise be viewed as an emerging platform rather than an established clinical rescue strategy (157).

### 5.4 Improving sequencing and treatment duration

Rational sequencing should account for antigen expression, disease kinetics, manufacturing feasibility, prior exposure and residual immune reserve. Bispecific antibodies can be delivered rapidly but repeatedly recruit the endogenous T cell pool; CAR-T cells require adequate leukapheresis quality and manufacturing time but provide a finite cellular intervention. Current evidence does not support a universal sequence, and prospective studies should measure how each therapy changes the fitness of the substrate required by the next treatment (161, 162).

Treatment duration is particularly relevant to bispecific antibodies. Continuous dosing may sustain response but also prolong immune suppression and repeated CD3 engagement. Experimental work supports partial recovery during treatment-free intervals, and finite-duration clinical approaches are being explored, but no validated biomarker currently defines when treatment can be safely stopped (141).

Sequential antigen targeting is another strategy. In myeloma, switching or sequencing BCMA-, GPRC5D- and FcRH5-directed therapies may address antigen escape and reduce selective pressure on a single tumor antigen. In B-cell malignancies, CD19- and CD20-directed strategies can be sequenced or combined conceptually, although optimal ordering remains disease-specific. Tumor debulking before T cell engagement may also preserve fitness by reducing antigen load and chronic stimulation; preclinical myeloma models have shown that high tumor burden can limit BCMA×CD3 bispecific antibody efficacy through T cell exhaustion (89).

Post-treatment immunomodulatory drugs require similar caution. IMiDs can enhance T cell and natural-killer-cell activity, and small nonrandomized studies have reported feasibility of lenalidomide after CAR-T cell therapy in selected lymphoma and myeloma populations (163, 164). However, increasing cytokine release is not itself a desirable endpoint. The relevant goal is durable antitumor function without exacerbating CRS, cytopenias, infection or delayed immune recovery. Evidence after bispecific antibodies is even more limited. IMiD maintenance or combination therapy should therefore be considered investigational or individualized within a clinical-trial framework rather than recommended routinely.

In summary, preservation should be prioritized before irreversible attrition occurs. Earlier collection, non-lymphotoxic disease control, modality-specific biomarker assessment, fitter product engineering and avoidance of unnecessary chronic stimulation are more defensible than attempting to activate a severely senescent or terminally exhausted compartment after treatment failure (**Figure 6**).

### 5.5 Clinical implementation: what can be assessed before treatment?

A pretreatment fitness assessment can support risk stratification, trial design and sample banking, but it should not currently be used as a stand-alone reason to deny an approved therapy. A pragmatic evaluation includes clinical context, accessible immune-cell measurements and, when feasible, functional testing. Interpretation must be modality-specific and should account for recent corticosteroids, chemotherapy, infection and tissue compartment. The proposed checklist below identifies variables worth testing prospectively; it does not establish validated eligibility thresholds (**Table 3**).

**Table 3:** Pragmatic pretreatment and longitudinal T cell fitness checklist.

Assessment domain	Before CAR-T cell collection/infusion	Before or during bispecific-antibody therapy
Clinical and treatment context	Prior lymphotoxic therapy, corticosteroid exposure, active infection, disease burden and tempo	Disease burden, infection history, immunoglobulins, cytopenias and prior T cell redirection
Cell quantity and phenotype	ALC, CD3/CD4/CD8 counts, viable collection yield, naïve/TSCM/TCM fractions, CD27/CD28 and CD57/KLRG1	CD3/CD4/CD8 counts, memory composition and serial change during treatment
Dysfunction and function	Checkpoint co-expression plus proliferation, cytokine production and killing when feasible	Checkpoint co-expression, proliferation/serial killing and longitudinal attrition
Product/kinetic measures	Viability, transduction, CD4:CD8 composition, expansion potential; post-infusion expansion and persistence	Not applicable to manufacture; monitor endogenous-cell phenotype, response and immune reconstitution
Disease- and tissue-specific factors	Marrow versus blood in MM/AML; tissue contexture in lymphoma; antigen density and escape risk	Tumor burden, target expression, marrow/lymph-node context and antigen switching

## 6 Conclusions and future perspectives

T cell-directed immunotherapy has transformed hematological oncology, but the next gains will depend on measuring and preserving the immune substrate as carefully as the tumor target. Relapse, limited persistence, immune-reconstitution failure and infection reflect both tumor biology and the state of the recruited or engineered T cells (165–167).

A central message of this Review is that T cell dysfunction in hematological cancer should not be reduced to exhaustion alone. Exhaustion, senescence and terminal differentiation are biologically related but mechanistically distinct (16). Exhaustion is largely driven by chronic antigen stimulation and stabilized by transcriptional and epigenetic programmes involving factors such as TOX and NR4A (168). Senescence reflects ageing, replicative history, telomere attrition, DNA damage responses and loss of proliferative capacity. Terminal differentiation reflects a cytotoxic but less plastic end stage of effector development. These states can coexist in the same patient, and even within related clonotypes, but they have different therapeutic implications (169). A checkpoint-high T cell may be reversibly exhausted, terminally exhausted, recently activated or senescent-like; therefore, single markers such as PD-1 or CD57 cannot be interpreted outside disease context, tissue niche and treatment history (170).

The clinical relevance of this distinction is already visible. In CAR-T cell therapy, product quality and cellular composition are critical. Fraietta and colleagues showed in chronic lymphocytic leukaemia that CAR-T cells from complete responders were enriched for memory-related gene programmes, whereas non-responders had T cells with programmes associated with effector differentiation, glycolysis, exhaustion and apoptosis; sustained remission was linked to a higher frequency of CD27^+^CD45RO^-^CD8^+^ T cells before CAR-T cell generation (17). In experimental CAR-T cell models, tonic signalling can induce early exhaustion, and 4-1BB co-stimulation can reduce exhaustion induced by persistent CAR signalling compared with CD28 co-stimulation in that setting (80). These observations support a broader principle: the engineered receptor is only one component of the therapy; the differentiation and fitness state of the cell carrying the receptor is equally important.

For bispecific antibodies, the key problem is different. These agents do not require ex vivo manufacture, but they depend on the patient’s remaining endogenous T cell pool. In multiple myeloma, single-cell and T cell receptor-tracing studies have shown that the pre-existing T cell landscape affects response to BCMA×CD3 T cell engagers; clonal expansion of effector CD8^+^ T cells can drive therapeutic activity, whereas an abundance of exhausted CD8^+^ clone predicts response failure (89). Continuous or repeated CD3 engagement may also become a source of immune pressure, particularly in patients with high tumor burden or already compromised immune reserves (89). Treatment-free intervals and fixed-duration approaches are therefore not merely clinical convenience strategies but also immunological hypotheses: they may reduce chronic stimulation and allow partial T cell functional recovery, although they require prospective validation.

Checkpoint blockade illustrates the limits of relying on inhibitory receptor expression alone. PD-1 blockade is highly active in selected hematological malignancies such as classical Hodgkin lymphoma; nivolumab received accelerated FDA approval in relapsed or progressive classical Hodgkin lymphoma after autologous transplantation and brentuximab vedotin, with a 65% objective response rate in the evaluated population. However, the failure and safety concerns of pembrolizumab combinations in multiple myeloma, including the FDA-required discontinuation of KEYNOTE-183 and KEYNOTE-185 owing to an increased risk of death when pembrolizumab was combined with immunomodulatory agents, demonstrate that PD-1 expression does not necessarily identify a reversibly exhausted, safely rescuable anti-tumor T cell population. Future checkpoint strategies in blood cancers will require sharper biomarkers of tumor-reactive, reversible dysfunction rather than broad assumptions based on PD-1 positivity (171).

MM remains a useful model because it combines immune aging, marrow suppression, repeated therapy and broad use of CAR-T cells and bispecific antibodies. However, the framework must not be generalized without modification: lymphoma requires tissue-contexture assessment, ALL requires persistence and antigen-escape monitoring, and AML requires simultaneous consideration of fitness and antigen selectivity (172). These developments support the broader question of whether earlier immune intervention can capture a fitter T cell compartment before irreversible attrition occurs. Priorities for prospective research are: standardized core phenotyping and functional assays; paired blood and tissue sampling where relevant; longitudinal measurement before and after each T cell-directed therapy; external validation of modality-specific predictive models; and integration of response, persistence, infection and immune-reconstitution endpoints. Real-world studies are essential for testing whether trial-derived biomarkers remain informative in older, frailer and more heavily pretreated populations (173–175).

Next-generation products—including gene-edited and armored CAR-T cells, allogeneic platforms, CAR-NK cells and metabolic interventions—may reduce dependence on compromised autologous cells, but they introduce new questions of persistence, rejection, lineage-specific safety and cost. These strategies should be evaluated against clearly defined fitness domains rather than a generic claim of “reversing exhaustion.” (176–178). In conclusion, T cell fitness should be treated as a measurable, disease- and modality-specific therapeutic substrate. Current evidence supports multidomain assessment and immune-preserving treatment design, but not a universal score or a single biomarker-based treatment rule. The immediate clinical priority is to collect and characterize cells at informative time points, avoid unnecessary lymphotoxic or inflammatory pressure, and test fitness-guided sequencing prospectively.
